# Peri-implant clinicoradiographic status among betel-quid chewers and controls

**DOI:** 10.1590/0103-6440202204676

**Published:** 2022-08-26

**Authors:** Montaser N Alqutub, Yasser Alali, Huda I. Tulbah, Fawad Javed, Fahim Vohra, Tariq Abduljabbar

**Affiliations:** 1 Department of Periodontics and Community Dentistry, College of Dentistry, King Saud University, Riyadh 11545, Saudi Arabia; 2 Department of Maxillofacial Surgery, College of Dentistry, King Saud University, RiyadhSaudi Arabia; 3 Department of Prosthetic Dental Sciences, College of Dentistry, King Saud University. Riyadh, Saudi Arabi;; 4 Department of Orthodontics, Eastman Institute for Oral Health, University of Rochester, NY, United States; 5 Department of Prosthetic Dental Sciences, College of Dentistry, King Saud University Riyadh 11545, Saudi Arabia

**Keywords:** Alveolar bone loss, Betel-quid, Crestal bone loss, Dental implant, Probing depth, Smokeless tobacco

## Abstract

The aim of the present case-control observational study was to evaluate the peri-implant clinicoradiographic status among betel-quid chewers and controls. Self-reported betel-quid chewers and controls were included. Participants were categorized into 3 groups: Group-1: Individuals chewing betel-quid with tobacco; Group-2: Individuals chewing betel-quid without tobacco; and Group-3: Controls (individuals not using tobacco in any form). Demographic data was collected using a questionnaire. Periodontal and peri-implant clinicoradiologic parameters (plaque and gingival indices [PI and GI], probing depth [PD] and crestal bone loss/marginal bone loss [CBL/MBL]) were assessed. Clinical attachment loss (AL) around teeth was also assessed. Group comparisons were done using the one-way analysis of variance and Bonferroni Post-hoc adjustment tests. Correlation of periodontal and peri-implant inflammatory parameters with the duration of betel-quid chewing habit and duration of placement in the mouth were assessed using logistic regression analysis. P<0.05 was considered statistically significant. Thirty, 30 and 30 patients were included in groups 1, 2 and 3, respectively. Full-mouth PI (P<0.01), GI (P<0.01), clinical AL (P<0.01), PD (P<0.01) and mesial and distal MBL (P<0.01) were higher in groups 1 and 2 than Group-3. Peri-implant mPI (P<0.01), mGI (P<0.01), PD (P<0.01) and MBL/CBL (P<0.01) were significantly higher in groups 1 and 2 than Group-3 with no significant difference in groups 1 and 2. Betel-quid chewing habit either with or without tobacco is a risk-factor of peri-implant soft-tissue inflammation and CBL.

## Resumo

O objetivo do presente estudo observacional de casos-controles foi avaliar o estado clínico-radiográfico periimplantar dos usuários de bétele. Foram incluídos usuários que relataram utlizar a substância bétele como tabaco de mascar. Os participantes foram categorizados em 3 grupos: Grupo-1: Indivíduos que mascam bétele com tabaco; Grupo-2: Indivíduos que mascam bétele sem tabaco; e Grupo-3: Controle (indivíduos que não usam tabaco sob qualquer forma). Os dados demográficos foram recolhidos utilizando um questionário. Foram avaliados parâmetros clínico-radiográfico e periimplantares (índices placa e gengivais [IP e IG], profundidade de sondagem [PS] e perda de crista óssea/ perda óssea marginal [PCO/POM]). Também foi avaliada a perda inserção clínica (IC) em torno dos dentes. As comparações de grupo foram feitas utilizando a análise de variância unidireccional e os testes de ajustamento post-hoc de Bonferroni. A correlação dos parâmetros inflamatórios periodontais e periimplantares com a duração do hábito de mastigação da bétele e duração da colocação na boca foi avaliada utilizando a análise de regressão logística. P<0,05 foi considerado estatisticamente significativo. Foram utilizados 30 pacientes em cada grupo. O IP de boca inteira (P<0,01), IG (P<0,01), IC clínica (P<0,01), PS (P<0,01) e POM mesial e distal (P<0,01) foram mais elevados nos grupos 1 e 2 do que no grupo 3. O mPI peri-implantar (P<0,01), ‘(P<0,01), PD (P<0,01) e POM/PCO (P<0,01) foram significativamente mais elevados nos grupos 1 e 2 do que no grupo 3, sem diferença significativa nos grupos 1 e 2. O hábito de mastigar a substância bétele com ou sem tabaco é um fator de risco de inflamação dos tecidos moles periimplantares e PCO.

## Introduction

Tobacco is addictive and is commonly smoked in the form of cigarettes, cigars, pipe and waterpipe and electronic nicotine delivery systems ^1^ ^,^ ^2^ ^,^ ^3^; and is a classical risk-factor for periodontal and peri-implant diseases ^4^ ^,^ ^5^. Over the years, numerous studies) ^6^ ^,^ ^7^ ^,^ ^8^ have confirmed that tobacco smoking jeopardizes the clinicoradiographic, microbiological and immunoinflammatory status of dental implants. However, many individuals consume tobacco in the form of smokeless tobacco (ST) products such as snuff, gutka and betel-quid. It is well documented that consumption of ST products is by no means a safe alternate to smoking and is associated with detrimental health hazards including periodontitis, oral submucous fibrosis (OSF), oral cancer, cardiovascular diseases and hepatic and renal toxicity ^9^ ^,^ ^10^ ^,^ ^11^ ^,^ ^12^.

**Figure 1:**
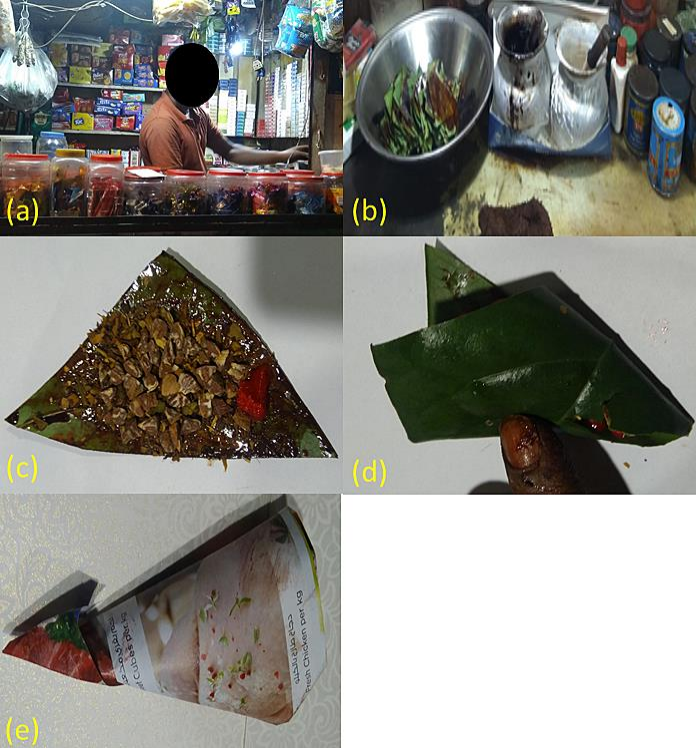
(a) Street-side betel-quid vendor; (b) Display of betel-quid ingredients including Piper betle leaf, slaked lime in pots, powdered tobacco and artificial fragrances in colorful tins; (c) betel-quid containing areca-nut, powdered tobacco, slaked lime and artificial sweetener/fragrance; (d) betel-quid folded over its constituents before being ingested or dispatched; (e) betel-quid dispatched in paper folded in a “kite-pattern”.

Betel-quid chewing is a social norm in Asian countries including Sri Lanka, India, China, Pakistan, and Nepal ^13^ ^,^ ^14^. However, this habit is also practiced by migrant communities residing in the United Kingdom, Italy and the United States ^15^ ^,^ ^16^ ^,^ ^17^. A betel-quid is composed of a variety of ingredients that are wrapped in the *Piper betle* leaf (PBL). These ingredients primarily comprise of areca nuts (AN), and aqueous pastes of calcium hydroxide paste or slaked lime (SL) and *Acacia catechu*. Both pastes are individually placed on the PBL in varying proportions and manually mixed; and AN and powdered tobacco are sprinkled over it. Other ingredients of betel-quid include powdered tobacco, artificial sweeteners, saffron, and menthol. There is no precise recipe for betel-quid preparation as the quantity of ingredients vary upon individual preference. The betel-quid is commonly and non-commercially sold by unlicensed street-side vendors (Figure 1a and 1b); however, it is also domestically prepared by users as the ingredients, such as PBL, AN, SL and powdered tobacco are commercially sold in public markets. The PBL is folded over its ingredients in a triangular pattern and dispatched in a paper usually folded in a “kite-pattern” (Figure 1c to 1e). The betel-quid is accessible to consumers of all age groups including school-going children and adolescents. ^18^ ^,^ ^19^ For consumption, the betel-quid is placed in the buccal vestibule and gently chewed following which, it is placed in the buccal vestibule (usually on the right side) for prolonged durations. The betel-quid is then continued to be gently chewed and sucked spasmodically. When desired, the betel-quid bolus is either swallowed or spat out. Habitual betel-quid usage has been linked with oral diseases including periodontitis and oral cancer. ^13^ ^,^ ^20^ It has been proposed that betel-quid chewing causes oral microbiome dysostosis, releases endotoxins and downregulates antioxidant proteins thereby leading to the formation and accumulation of reactive-oxygen-species (ROS) in tissues ^21^. This proposed mechanism has been linked with oral inflammatory conditions including carcinogenesis ^21^. Javed et al. ^13^ assessed periodontal inflammation in controls and individuals chewing betel-quid with and without tobacco. The results showed that gingival bleeding, and increased probing depth (PD) and marginal bone loss (MBL) were significantly higher in patients that chewed betel-quid with than without tobacco and controls ^13^. Similar results were reported in clinical studies by Akhter et al. ^22^ and Hsiao et al. ^23^ These results indicate that betel-quid chewing habit is a risk-factor of periodontitis, which in turn is linked with the etiopathogenesis of peri-implant diseases including peri-implantitis ^24^. A thorough review of indexed literature revealed no studies that assessed the clinicoradiographic status of dental implants in betel-quid chewers. The authors hypothesize that peri-implant clinical (modified plaque index [mPI] and modified gingival indices [mGI], respectively) and PD) and radiographic (crestal bone loss [CBL]) parameters are aggravated among individuals chewing betel-quid compared with controls.

The present study evaluated the peri-implant clinicoradiographic status among betel-quid chewers and controls (individuals not using any form of nicotinic product).

## Material and methods

### Ethical statement

Guidelines recognized by the Declaration of Helsinki as revised in 2013 for experimentation involving human patients were followed. All volunteering individuals were requested to read and sign a consent form. All participants were informed that they could withdraw their participation at any phase of the study without consequences. Ethical approval was obtained from ethics research committee of Centre for specialist dental practice and clinical research (UDCRC/025-16).

### Location

The study was performed at the Division of Periodontology and Implant Dentistry of a local oral healthcare center located in Riyadh, Saudi Arabia. The study was conducted between February 2020 and November 2020.

### Study design and eligibility criteria

In the present cohort study, self-reported betel-quid chewers (individuals chewing at least one betel-quid daily for at least 1 year) and controls (individuals not using nicotinic products) were included.^25^ Patients having under gone dental implant therapy for partial edentulism were included. Self-reported immunosuppressed individuals (such as patients with cancer, diabetes mellitus [DM], acquired immune deficiency syndrome/HIV, cardiovascular diseases, and hepatic and renal diseases) were excluded. Tobacco-smokers and individuals using ST products other than betel-quid such as *gutka*, snuff and *shamma* were not sought. Furthermore, individuals that reported to have undergone chemo- and/or radiotherapy, probiotic and/or antibiotic therapy, or had used non-steroidal anti-inflammatory medications within the past 2-months were excluded. Dental implants placed in grafted sites, third molars and grossly-carious teeth were not assessed

### Study groups

With reference to betel-quid chewing habit, participants were categorized into the following groups: Group-1: Individuals chewing betel-quid with tobacco; Group-2: Individuals chewing betel-quid without tobacco; and Group-3: Individuals that reported to have never used tobacco in any form (controls).

### Demographics and betel-quid chewing related information

Information regarding age in years, gender (male/female/prefer not to respond), betel-quid chewing (yes/no), betel-quid chewing with tobacco (yes/no), duration of betel-quid chewing habit in years, family history of betel-quid chewing (yes/no), daily toothbrushing (once/twice/3x times or more), daily flossing (once/twice/3x or more), buccal vestibule in which, the betel-quid is placed (right, left or both), duration for which, each betel-quid was placed in the buccal vestibule, and amount of tobacco (in grams) added to each betel-quid. A trained investigator administered the questionnaire to all participants.

### Dental implants

One investigator explored patients’ dental records and recorded the following data (a) duration of implants in function in years; (b) implant surface characteristics; (c) implant abutment junction (platform or non-platform switched); (d) implant length and diameter; (e) jaw location; (f) implant prosthesis retention (cement or screw retention); and (g) depth or placement (bone-level/crestal or sub-crestal).

### Clinical and radiologic examinations

In all patients, full mouth periodontal and peri-implant clinical and radiologic peri-implant were carried out as described elsewhere ^26^. In summary, plaque index (PI) ^27^, gingival index (GI) ^28^, clinical attachment loss (AL) ^29^ and PD ^30^ were assessed at 6-sites (distobuccal, mesiobuccal, mid-buccal, distolingual/palatal, mid-lingual/palatal, and mesiolingual/palatal) per tooth and implant. a graded probe (Hu-Friedy) was used to record the clinical AL and PD to the nearest millimeter.^5^ Bitewing radiographs (Ektaspeed plus; Kodak) were taken and viewed on a calibrated computer screen (Samsung SyncMaster digital TV monitor) using a software program (Image Tool 3.0, Department of Dental Diagnostic Science, University of Texas Health Science Center). The MBL was defined as the linear distance from 2 mm below the cementoenamel junction to the alveolar crest; ^5^ and CBL was defined as the vertical distance from 2mm below the implant abutment interface to crestal bone ^31^.

### Statistical and power analyses

Statistical analysis was performed using a software program (SPSS v.18, IBM). Group comparisons were done using the one-way analysis of variance and Bonferroni Post-hoc adjustment tests. Correlation of periodontal and peri-implant inflammatory parameters with the duration of betel-quid chewing habit and duration of placement in the mouth were assessed using logistic regression analysis. Power analysis was done using a software program (G*Power version 3.1.5., University of Kiel) on data obtained from a pilot study. Power analysis was performed for detecting MBL and CBL (primary outcome variables) in the study groups. It was estimated that with inclusion of at least 30 individuals per group, the study would attain a power of 91.5%. P-values below 0.05 were designated as statistically significant.

## Results

### Demographic results

Ninety patients (30, 30 and 30 in groups 1, 2 and 3, respectively) were included. In each group, most of the participants were male. There was no statistically significant difference in age among patients in all groups. In groups 1 and 2, individuals were chewing betel-quid for 14.5 ± 3.8 and 11.6 ± 1.3 years, were placing the quid in the right buccal vestibule and were consuming 9.2 ± 0.5 and 8.9 ± 0.2 quids/day. In groups 1 and 2, each betel-quid was placed in the mouth for a mean duration of 32.6 ± 5.6 and 23.5 ± 0.7 minutes, respectively. There was no significant difference in the duration of betel-quid placement in the mouth among patients in groups 1 and 2. None of the participants in Group-1 were aware of the average quantity (in grams) of powdered tobacco present in each betel-quid. A family history of betel-quid chewing was more often reported by individuals in groups 1 (70%) and 2 (60%) compared with Group-3 (30%). Toothbrushing twice daily was more often reported by individuals in Group-3 (73.3%) compared with individuals in groups 1 and 2, 23.3% and 33.3%, respectively. None of the individuals reported to have ever used a dental floss (Table 1).

**Table 1:** Characteristics of the study cohort

Parameters	Group-1	Group-2	Group-3
Participants (n)	30	30	30
Males : Females	21 : 9	22: 8	20 : 10
Age in years			
All patients	47.4 ± 5.2 years	45.1 ± 2.2 years	44.2 ± 1.3 years
Males	50.3 ± 3.7 years	48.4 ± 3.1 years	46.4 ± 0.6 years
Females	44.6 ± 2.7 years	41.8 ± 0.8 years	40.5 ± 2.4 years
Number of betel-quids consumed daily			
All patients	9.2 ± 0.5 quids/day	8.9 ± 0.2 quids/day	NA
Males	9.6 ± 2.1 quids/day	8.7 ± 0.4 quids/day	NA
Females	8.6 ± 1.5 quids/day	9.3 ± 0.7 quids/day	NA
Duration of betel-quid chewing habit (in years)			
All patients	14.5 ± 3.8 years	11.6 ± 1.3 years	NA
Males	15.1 ± 2.6 years	14.6 ± 1.9 years	NA
Females	12.7 ± 3.1 years	11.7 ± 0.4 years	NA

**Table 1:** Continuation.

Parameters	Group-1	Group-2	Group-3
Buccal vestibule in which the quid is placed			
Right	30	30	NA
Left	None	None	NA
Duration of each betel-quid placement in the mouth (in minutes)			
All patients	32.6 ± 5.6 minutes	23.5 ± 0.7 minutes	NA
Males	37.3 ± 2.8 minutes	25.2 ± 1.7 minutes	NA
Females	30.4 ± 1.5 minutes	16.8 ± 0.5 minutes	NA
Quantity of tobacco in each quid (in grams)			
All patients	NR	NA	NA
Males	NR	NA	NA
Females	NR	NA	NA
Family history of betel-quid chewing	21 (70%)	18 (60%)	9 (30%)
Toothbrushing			
Once daily	23 (76.7%)	20 (66.7%)	8 (26.7%)
Twice daily	7 (23.3%)	10 (33.3%)	22 (73.3%)
3x or more	0	0	0
Interproximal flossing	0	0	0

NA: Not applicable NR: Not reported Group-1: Individuals chewing betel-quid with tobacco Group-2: Individuals chewing betel-quid without tobacco Group-3: Individuals not using tobacco in any form

### Dental implants

In all groups, the implants were platform-switched with moderately rough surfaces. The lengths and diameters of the implants ranged between 11-14 mm and 4.1-4.8 mm, respectively. All implants were placed at bone-level and prosthetic loading had been done using screw-retained restorations.

Thirty-one, 33 and 32 single-unit platform switched implants with moderately rough surfaces were present among patients in groups 1, 2 and 3. Most of the implants were located in the areas of missing mandibular right first and/or second molars in all groups. In groups 1, 2 and 3, the implants were in function for 3.6 ± 0.2, 3.3 ± 0.3 and 3.4 ± 0.3 years, respectively (Table 2). All implants located in the areas of missing maxillary and mandibular first or second molars; and were placed by a trained and experienced oral surgeon using insertion torques ranging between 30 and 35 Ncm. All implants were placed at bone level in healed sites. The lengths and diameters of the implants ranged between 11, 13, 4, and 4.1 mm, respectively. All implants were restored with cement-retained restorations.

**Table 2:** Characteristics of implants

Parameters	Group-1	Group-2	Group-3
Number of implants (n)	31	33	32
Mandibular right^*^	27	25	26
Mandibular left^*^	1	3	2
Maxillary right^*^	1	3	3
Maxillary left^*^	2	2	1
Duration of implants in function in years	3.6 ± 0.2 years	3.3 ± 0.3 years	3.4 ± 0.3 years

Group-1: Individuals chewing betel-quid with tobacco; Group-2: Individuals chewing betel-quid without tobacco Group-3: Individuals not using tobacco in any form.

* Missing molars

### Periodontal and peri-implant clinical-radiographic status

Full-mouth PI (P<0.01), GI (P<0.01), clinical AL (P<0.01), PD (P<0.01) and mesial and distal MBL (P<0.01) were significantly higher among patients in groups 1 and 2 compared with Group-3. There was no statistically significant difference in full-mouth PI, GI, clinical AL, PD and mesial and distal MBL among patients in groups 1 and 2 (Table 3). Peri-implant mPI (P<0.01), mGI (P<0.01), PD (P<0.01) and mesial and distal CBL (P<0.01) were significantly higher among patients in groups 1 and 2 compared with Group-3. There was no statistically significant difference in peri-implant mPI, mGI, PD and mesial and distal CBL among patients in groups 1 and 2 (Table 4).

**Table 3:** Full mouth clinicoradiographic periodontal status

Parameters	Group-1	Group-2	Group-3
Plaque index	2.6 ± 0.3^*^	2.1 ± 0.2^*^	0.4 ± 0.05
Gingival index	2.8 ± 0.4^*^	2.5 ± 0.2^*^	0.5 ± 0.08
Clinical AL	3.5 ± 0.05 mm^*^	3.2 ± 0.2 mm^*^	0.6 ± 0.04 mm
Probing depth	4.7 ± 0.2 mm^*^	4.3 ± 0.2 mm^*^	1.4 ± 0.03 mm
Marginal bone loss (mesial)	4.4 ± 0.2 mm^*^	4.5 ± 0.3 mm^*^	1.5 ± 0.06 mm
Marginal bone loss (distal)	4.5 ± 0.06 mm^*^	4.2 ± 0.05 mm^*^	1.3 ± 0.02 mm

Group-1: Individuals chewing betel-quid with tobacco; Group-2: Individuals chewing betel-quid without tobacco; Group-3: Individuals not using tobacco in any form.

* There was a statistically significant difference when compared with Group-3 (P<0.01)

**Table 4:** Peri-implant clinicoradiographic status

Parameters	Group-1	Group-2	Group-3
Plaque index	2.5 ± 0.08^*^	2.3 ± 0.1^*^	0.4 ± 0.06
Gingival index	3.1 ± 0.04^*^	2.7 ± 0.07^*^	0.2 ± 0.005
Probing depth	4.6 ± 0.2 mm^*^	4.4 ± 0.3 mm^*^	1.5 ± 0.008 mm
Crestal bone loss (mesial)	3.7 ± 0.06 mm^*^	3.3 ± 0.1 mm^*^	0.4 ± 0.005 mm
Crestal bone loss (distal)	3.5 ± 0.08 mm^*^	3.4 ± 0.04 mm^*^	0.3 ± 0.007 mm

Group-1: Individuals chewing betel-quid with tobacco; Group-2: Individuals chewing betel-quid without tobacco; Group3: Individuals not using tobacco in any form. ^*^There was a statistically significant difference when compared with Group-3 (P<0.01)

Correlation of periodontal and peri-implant PD and CBL/MBL between duration of betel-quid chewing habit and duration of betel-quid placement in the mouth

There was no statistically significant correlation between duration of betel-quid chewing habit, daily frequency of betel-quid consumption and duration of betel-quid placement in the mouth with periodontal and peri-implant clinical-radiographic parameters among patients in groups 1 and 2 (data now shown).

## Discussion

Betel-quid Chewing is a complex behavior, which remains unaddressed in the field of clinical implant dentistry and associated research. To the authors knowledge, this is the first study that assessed the clinical and radiologic peri-implant parameters among betel-quid chewers and controls. The present study was based on the hypothesis that peri-implant clinical (mPI, mGI, and PD) and radiographic (CBL) parameters are intensified in individuals chewing betel-quid compared with controls. The present results are in accordance with the proposed hypothesis as the aforementioned parameters were poorer among patients habitually chewing betel-quid compared with controls. It is pertinent to note that periodontal and peri-implant inflammatory conditions were poorer in all betel-quid chewers compared with controls and this relationship was independent of whether or not powdered tobacco was added to the betel-quid. In a recent experimental study on rats, Al-Tayar et al.^32^ assessed the cytotoxic effects of betel-quid and AN-extract on mouse fibroblasts and human epithelial cell lines. The results showed that AN as well as betel-quid extracts (irrespective of their concentrations) significantly reduced cell-viability of fibroblasts and epithelial cells ^32^. The AN is a major constituent in the betel-quid and has been associated with an increased release of destructive-inflammatory cytokines from various immune dells; and has been linked with the etiopathogenesis of various oral and systemic hazards such as OSF and type-2 diabetes mellitus, obesity, cirrhosis, and epilepsy, respectively ^33^ ^,^ ^34^ ^,^ ^35^ ^,^ ^36^ ^,^ ^37^. The present results showed no significant correlation between duration of betel-quid chewing habit, daily frequency of consumption and duration of placement of the quid in the mouth and occurrence of peri-implant inflammatory conditions. This suggests that there is no minimum frequency of duration of chewing habit or minimum frequency of betel-quid consumption that may be considered safe in terms of induction or progression of periodontal and peri-implant diseases. The authors applaud results from previous studies ^38^ ^,^ ^39^ which have shown that betel-quid either with or without tobacco increases the risk of oral malignant and premalignant lesions. Based upon the present results, the authors propose that betel-quid chewing habit with or without tobacco increases the risk of periodontal and peri-implant diseases. It is noteworthy that dental implants were majorly located at the right side of mandible in all groups. The mere justification that we can provide in this context is that since participants were mostly using the right side for chewing food, it is likely that this side was more subjected to occlusal masticatory forces and cariogenic food items compared with the left side. These factors could be related to missing molars on the right than the left side. However, assessment of dietary patterns was beyond the scope of the present investigation.

An alarming result of the present study was that none of the individuals chewing betel-quid with tobacco (Group-1) were aware of the amount (in grams) of powered tobacco they were chewing along with the quid. While addressing the question about the quantity of tobacco chewed with the quid, nearly all individuals in Group-1 verbally responded either “not much” or “little bit” and left the section blank in the questionnaire. From an ethical perspective, none of the participants were obligated to respond to this or any of the questions. Based upon the present results, t is evident that individuals consuming betel-quid were unaware of the amount of tobacco they were using with each quid on a daily basis. Moreover, the present results showed that family history of betel-quid chewing was more often reported by individuals in groups 1 (70%) and 2 (60%) compared with Group-3 (30%). It is therefore important to educate the community about the detrimental effects of ST tobacco products (including betel-quid chewing) on oral as well as systemic health statuses. Community-based health education programs and anti-tobacco campaigns may play an essential role in this regard.

One strength of the present investigation is that to the authors’ knowledge this is the first power-adjusted study that compared the peri-implant clinical-radiographic status in betel-quid chewers and controls. However, there are a number of limitations associated with the present study. In the present study, subgingival microbiota was not assessed in the study groups. According to Islam et al. ^21^ betel-quid disrupts the oral microbiota and participates in carcinogenesis by producing ROS and endotoxins. Moreover, it was challenging to precisely determine the quantity of components such as areca-nut, powdered tobacco (PT) and slaked-lime consumed with each betel-quid. The authors hypothesize that ST products imbalances the microbial ecosystem in the oral biofilm thereby enhancing the growth of pathogenic bacteria (such as red complex bacteria) in the oral biofilm that aggravates peri-implant soft tissue inflammation and CBL. It is also anticipated that the quantity of areca-nut, slaked lime and PT influences the severity of peri-implant inflammation in betel-quid chewers. Furthermore, the present study had an observational cohort design. In this regard, the present study did not focus on the treatment of peri-implant diseases in betel-quid chewers. Nicotine (a major addictive component in tobacco) compromises or delays cutaneous and mucosal wound healing ^40^ ^,^ ^41^; therefore, it is anticipated that the outcomes of therapeutic regimes used for the management of peri-implant diseases (surgical and non-surgical mechanical debridement [MD]) are compromised in betel-quid chewers compared with controls; nevertheless, to date there is a lack of consensus whether surgical MD is superior to non-surgical MD or vis versa for the management of peri-implant diseases.^42^ Studies ^43^ ^,^ ^44^ have shown that betel-quid chewing produces a variety of subtle effects, including hyperthermia and increased pulse rate, heightening alertness and concentration, staving off hunger, lifting mood and sensation of wellbeing. However, the reasons for chewing betel-quid were not assessed in the present study. It is speculated that the reasons for betel-quid chewing habit are comparable among patients with periodontal and peri-implant diseases. Further studies are needed to test these hypotheses.

## Conclusions

Betel-quid chewing habit either with or without tobacco is a risk-factor of peri-implant soft-tissue inflammation and CBL.
